# Supplementary material for: Gut microbial signatures expose the westernized lifestyle of urban Ethiopian children
Source: Commun Biol. 2026 Jan 31;9:346. doi: 10.1038/s42003-026-09639-2 (PMC12963580; doi:10.1038/s42003-026-09639-2)
Supplement: Supplementary file 1 — Supplementary Information [file 42003_2026_9639_MOESM1_ESM.pdf]

Supplementary Information

Gut microbial signatures expose the westernized lifestyle of urban Ethiopian children

Lydia Kirsche,[1,*] Peter Leary,[1,2] Martin J. Blaser,[3] Michael Scharl,[4] Adugna Negussie[5] and Anne Müller[1,*]

[1]Institute of Molecular Cancer Research, University of Zürich, Zürich, Switzerland

[2]Functional Genomics Center Zürich, University of Zürich and ETH Zürich, Zürich, Switzerland

[3]Center for Advanced Biotechnology and Medicine, Rutgers University, Piscataway, NJ, USA

[4]Department of Gastroenterology and Hepatology, University Hospital Zurich, University of Zurich, Zurich, Switzerland

[5]Department of Medical Laboratory Sciences, College of Health Sciences, Arsi University, Asella, Ethiopia

[*]**Correspondence to**: Lydia Kirsche or Anne Müller, Institute of Molecular Cancer Research, University of Zürich, Winterthurerstr. 190, 8057 Zurich, Switzerland. Phone: +41 44 635 3474; Fax: +41 44 635 3484; kirsche@imcr.uzh.ch, mueller@imcr.uzh.ch

Published in *Communications Biology 2026*

Supplementary Figure 1

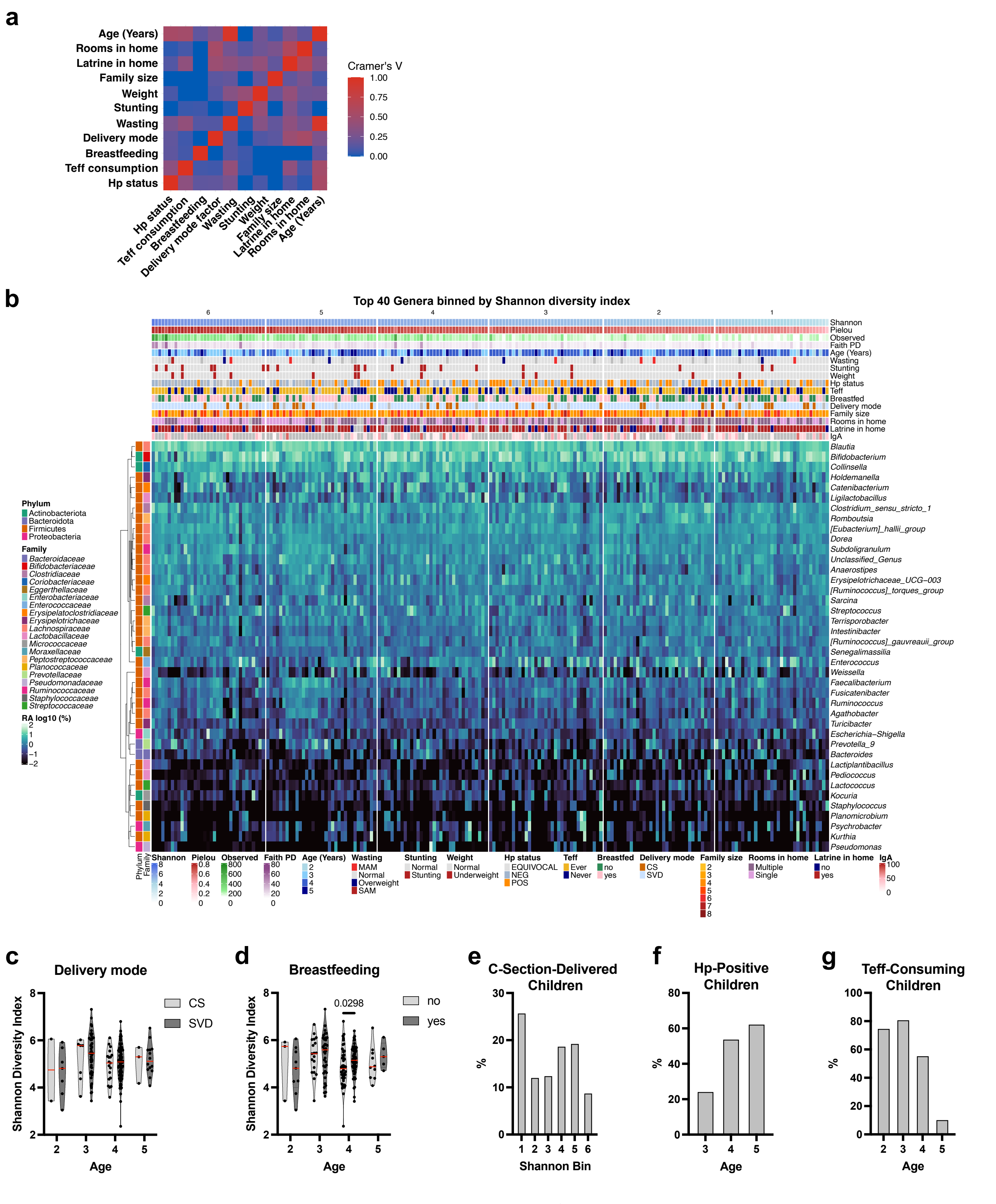

Supplementary Figure 1. Microbiome diversity and its early-life and environmental determinants in Ethiopian children. (a) Heatmap of Cramer's V (0-1) for pairwise associations between categorical variables. Values >0.7 indicate redundancy. **(b)** Heatmap of the top 40 genera identified by 16S rRNA gene sequencing. The heatmap shows log_{10}-transformed relative abundances (RA), with annotation at the genus level and colored by their respective phylum and family on the left. Annotations include sample metadata (sex, breastfeeding status, *Helicobacter pylori* (Hp) status, latrine availability, delivery mode, family size, consumption of products made from teff, anthropometric measures),

alpha diversity metrics (Shannon index, Pielou's evenness, observed features, Faith's phylogenetic diversity), and IgA-coating percentages where available. Samples are binned according to their Shannon diversity index, into six bins of approximately equal size (quantile-based binning), labelled 1-6 from lowest to highest. **(c)** Shannon diversity index plotted against age, stratified by delivery mode (CS: caesarean section; SVD: spontaneous vaginal delivery). **(d)** Shannon diversity index plotted against age, stratified by breastfeeding status (breastfed ≥18 months: yes or no). Statistical significance was assessed using Wilcoxon rank-sum tests between age groups. Red lines indicate medians. **(e)** Proportion of caesarean-delivered children across Shannon diversity bins, with the highest frequency observed in the lowest diversity bin. **(f)** Frequency of *H. pylori*-positive children aged 3-5 years, indicating an increase in *H. pylori* prevalence with age. **(g)** Frequency of teff consumption among children aged 2-5 years. All analyses include n=207 children.

Supplementary Figure 2

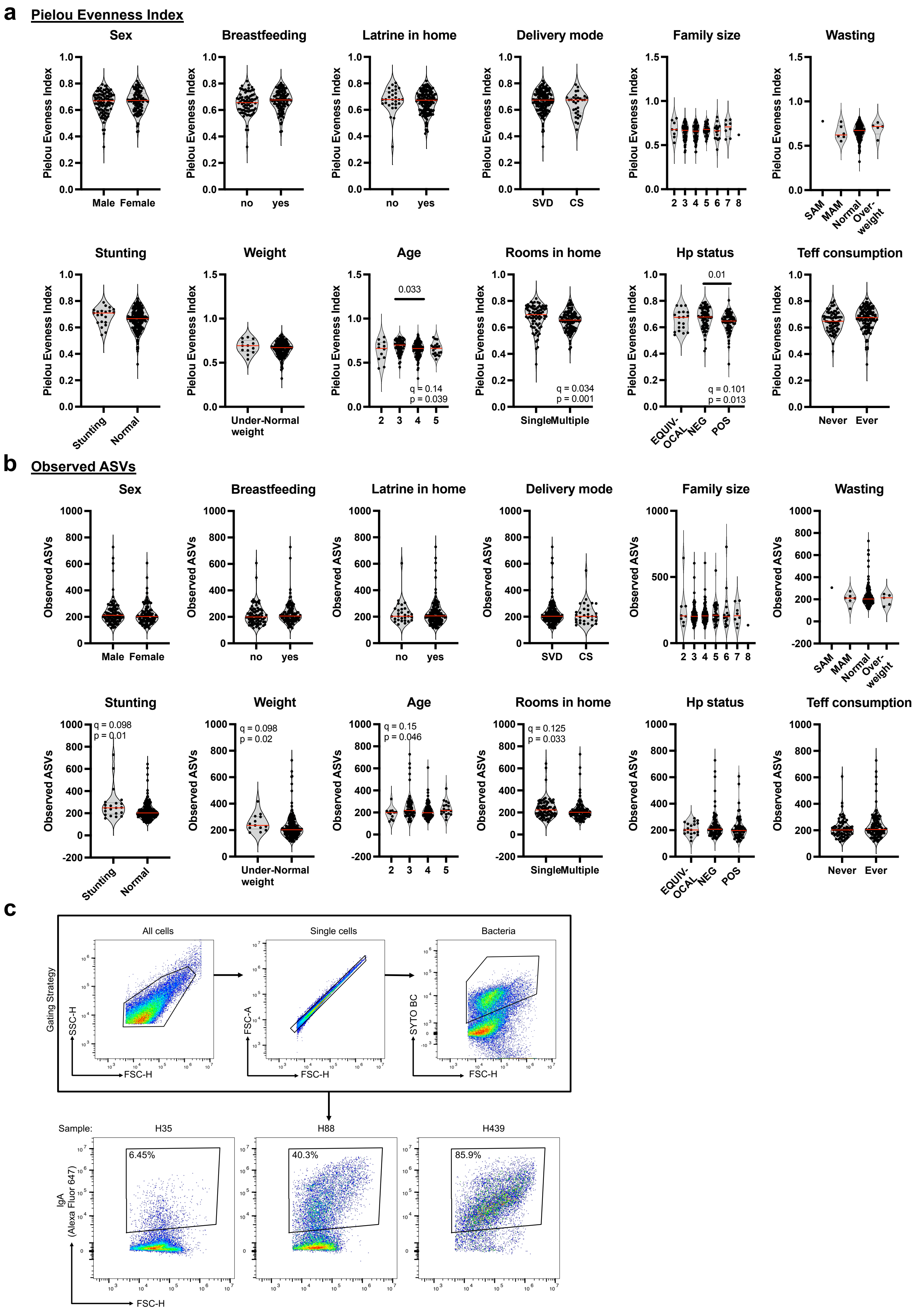

Supplementary Figure 2. Alpha-diversity metrics and IgA coating of the gut microbiota. (a) Pielou's evenness index plotted for all factors shown in the main figure 1. **(b)** Number of observed ASVs plotted for all factors shown in the main figure 1. Statistical significance was assessed using Wilcoxon rank-sum or Kruskal-Wallis tests with post-hoc testing and BH correction where appropriate. Only results with $p \leq 0.05$ are displayed in the figure, alongside the corresponding q-value (n=207). Red lines indicate medians. **(c)** Flow cytometry gating strategy and representative dot plots for IgA coating. The gating strategy (top) was applied to all samples. Below, representative dot plots illustrate low (6.45%), intermediate (40.3%), and high (85.9%) IgA coating. IgA (Alexa Fluor 647) is plotted against FSC-H after pregating on Syto BC^+ bacteria.

Supplementary Figure 3

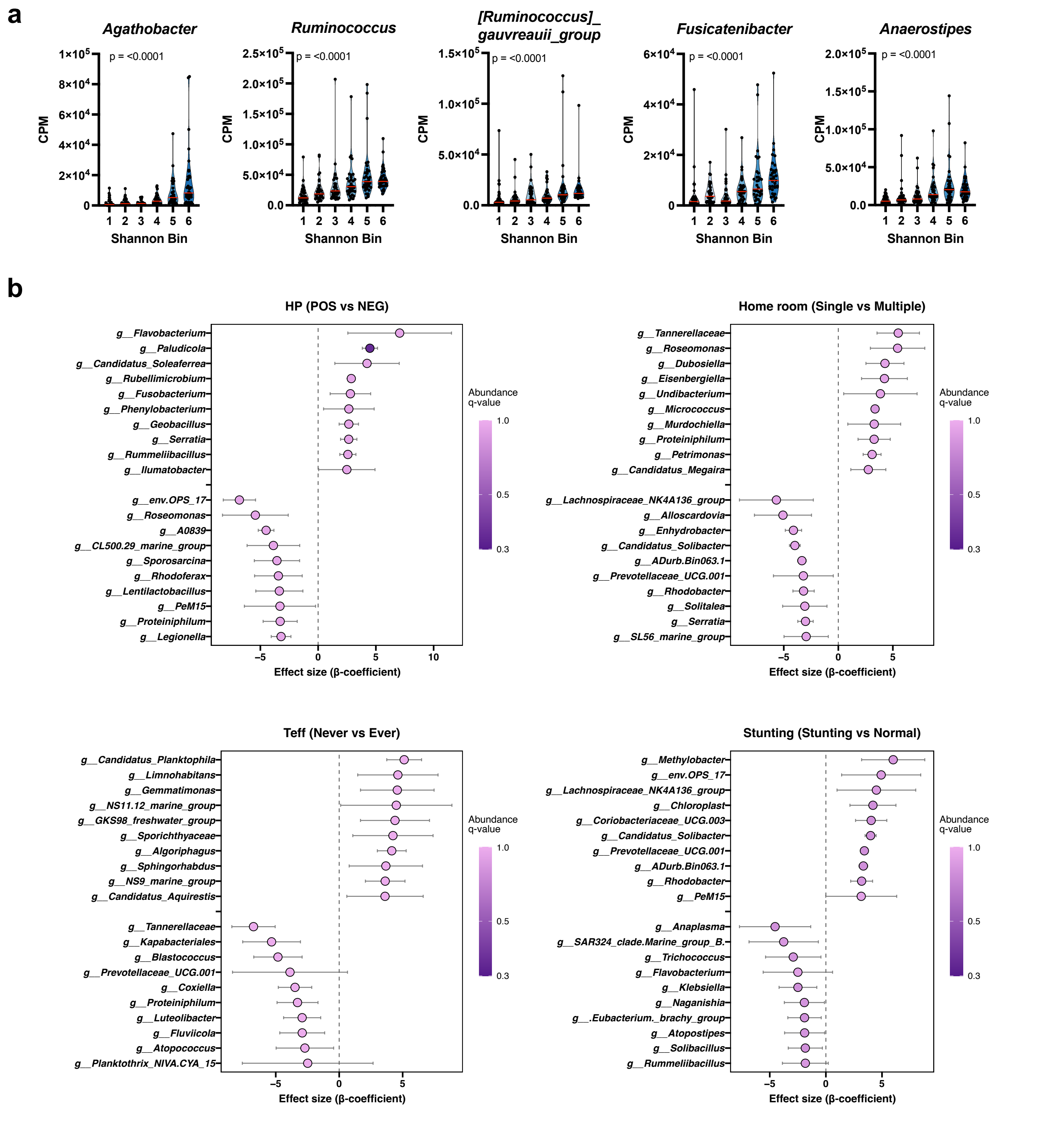

Supplementary Figure 3. Associations between bacterial genera, microbial diversity, and host or environmental factors. (a) Relative abundance (counts per million, CPM) of *Agathobacter*, *Ruminococcus*, *Ruminococcus_gauvreauii_group*, *Fusicatenibacter* and *Anaerostipes* are shown in relation to Shannon diversity bins. Associations were tested using linear regression models including Shannon diversity and age. The p-values from the regression models are plotted. Red lines indicate medians. **(b)** Top 10 genera most strongly positively and negatively associated (20 in total) with *H. pylori* infection status, household room number, teff consumption frequency, and stunting, as identified by MaAsLin3. Associations are shown at the genus level, with corresponding FDR-adjusted p-values and effect sizes (β-coefficients). Error bars indicate the standard error of the β-coefficients. All analyses include n=207 children.

Supplementary Figure 4

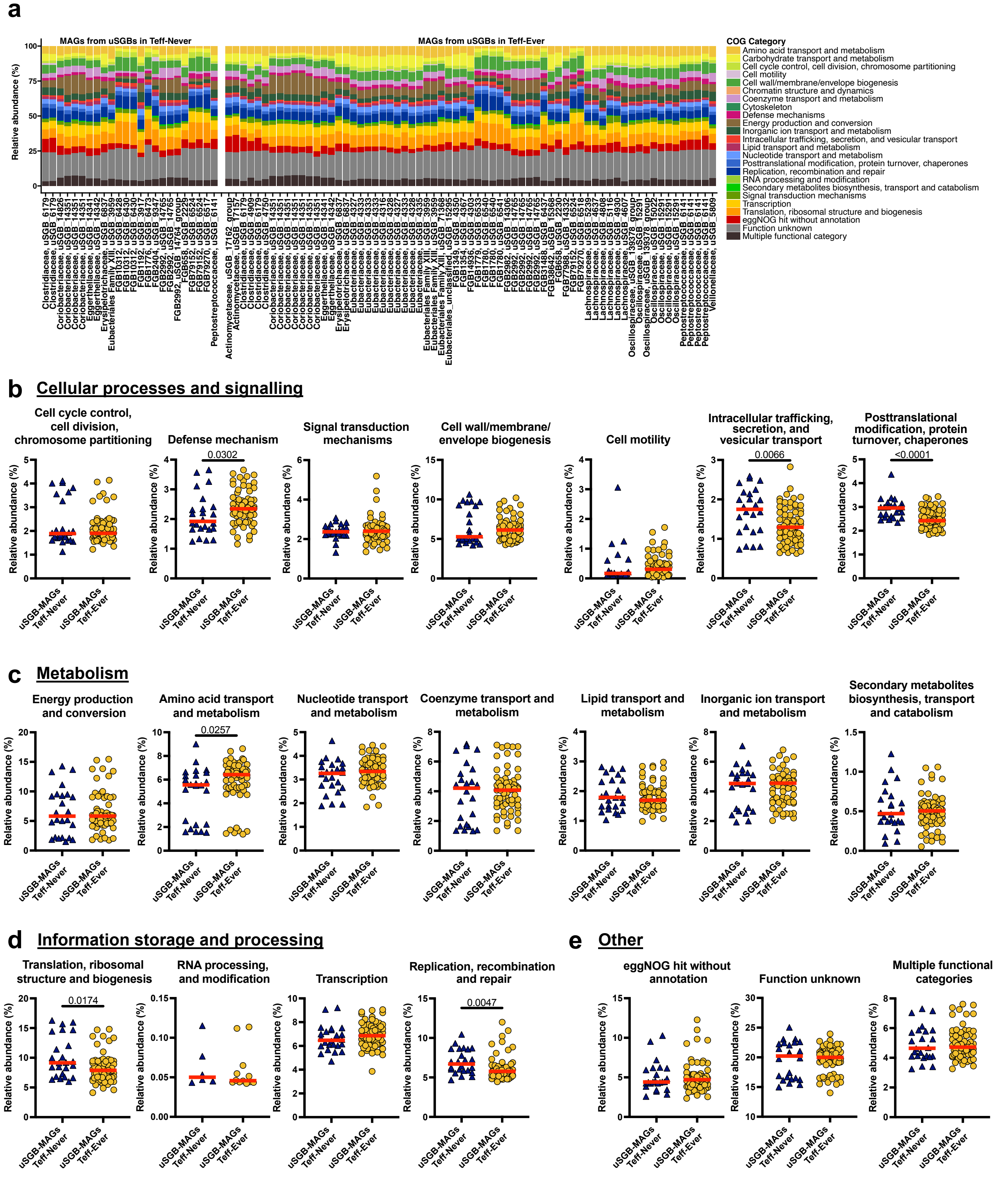

Supplementary Figure 4. Functional annotation of uSGBs of urban Ethiopian children. (a) Bar plot of the relative abundances of COG functional categories identified in the MAGs assigned to uSGBs from teff-consuming and non-teff-consuming children. Samples are labelled by uSGB and family-level taxonomic classification. Relative abundances are calculated per genome. **(b-e)** Relative abundance of COG functional categories in MAGs assigned as uSGBs in teff-consuming children versus non-consuming children. COG categories are grouped into major functional classes. Statistical significance was assessed using Wilcoxon rank-sum tests with BH correction. Red lines indicate medians. A total of 91 uSGBs derived from 105 children were included in the analysis.

Supplementary Figure 5

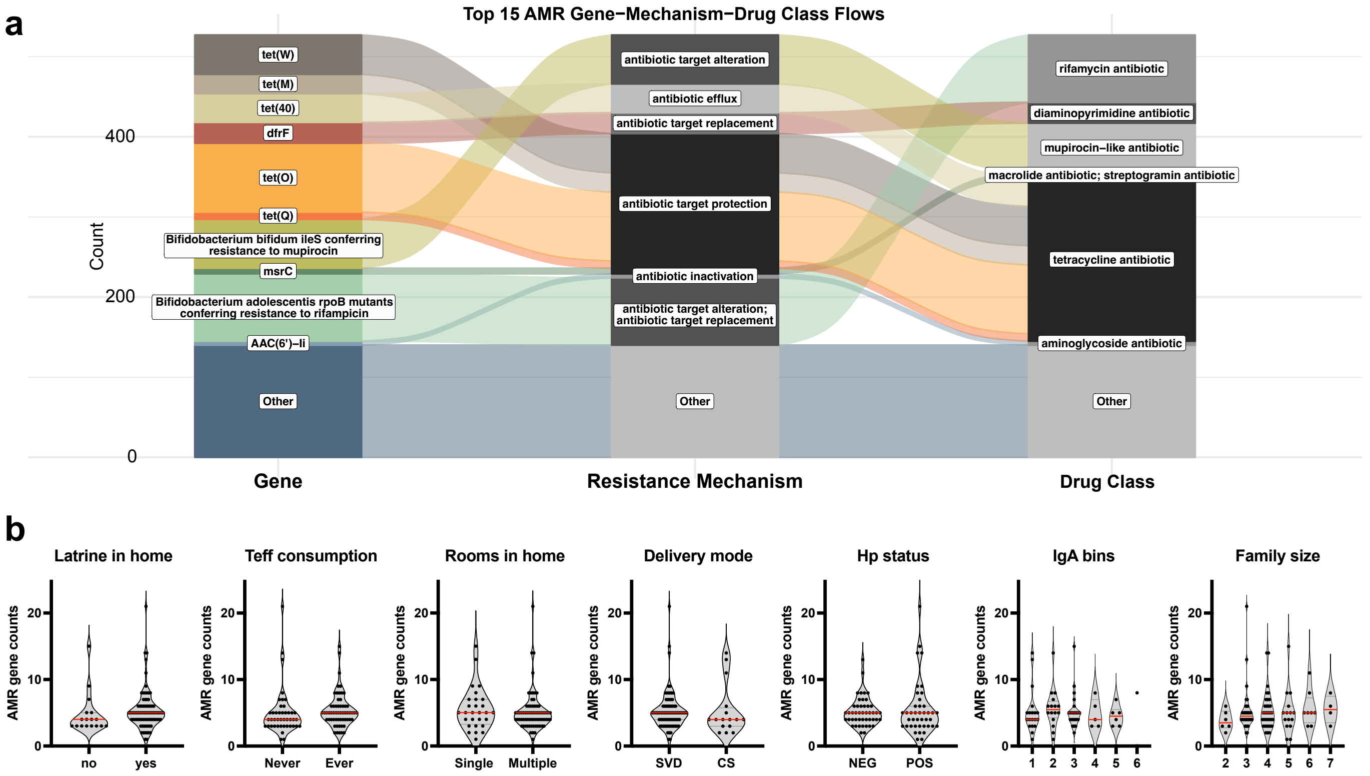

Supplementary Figure 5. Antimicrobial resistance profiles and functional associations in urban Ethiopian children. (a) Alluvial plot showing the top 10 antimicrobial resistance (AMR) genes, their associated resistance mechanisms, and drug classes identified from metagenomic sequencing data. **(b)** AMR gene counts stratified by key factors: presence of a latrine in the home, teff consumption, number of rooms in the home, delivery mode, *H. pylori* infection status, IgA-coating diversity bins and family size. Statistical significance was assessed using Wilcoxon rank-sum or Kruskal-Wallis tests with post-hoc testing and BH correction where appropriate. Red lines indicate medians. IgA-coating analyses include n=72 children; all other stratifications include n=105 children.

Supplementary Figure 6

a

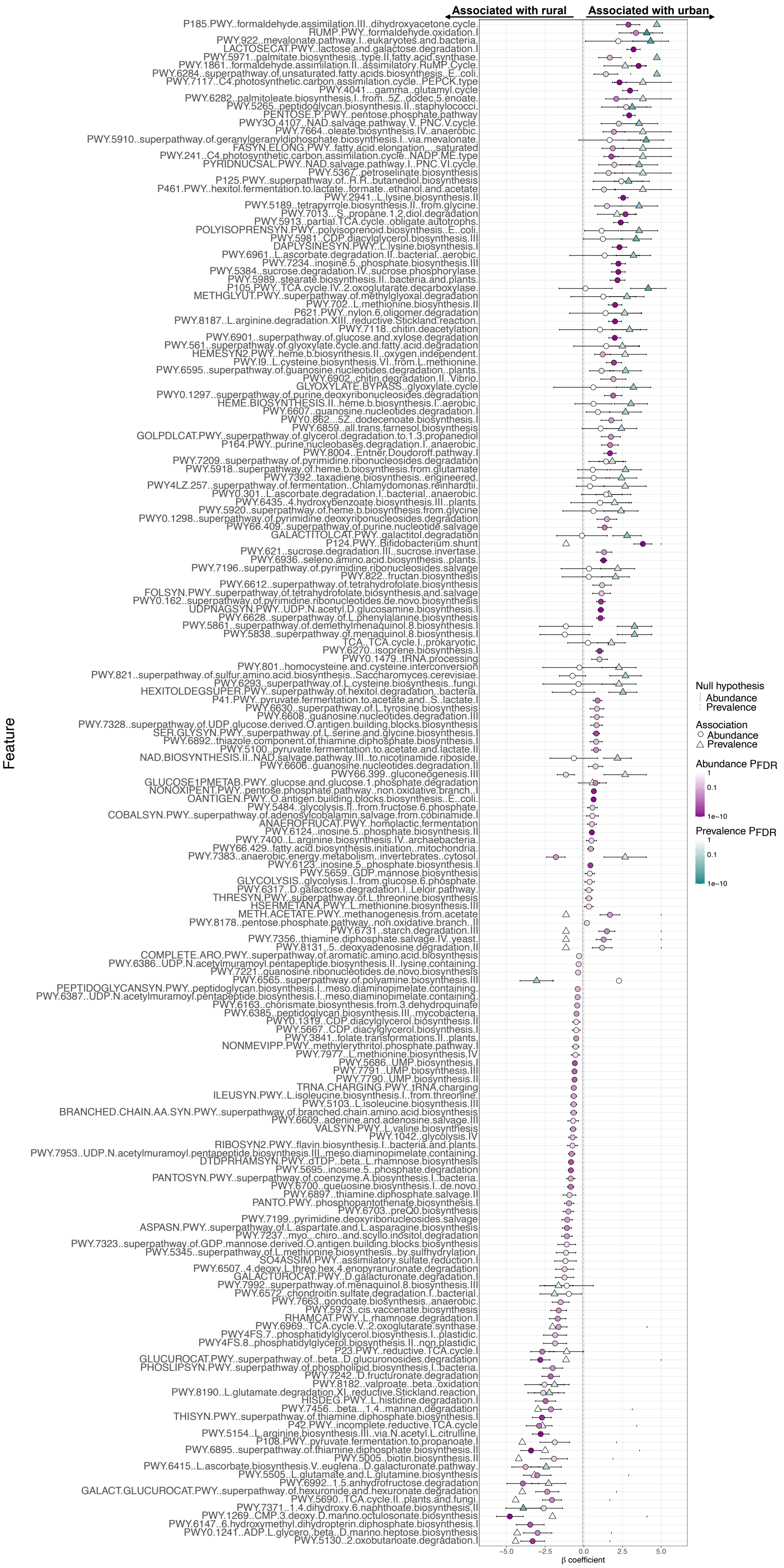

Supplementary Figure 6. Pathway associations with urban and rural origin. (a) Coefficient plot of pathway associations identified by MaAsLin3, displaying β-coefficients for both prevalence and abundance models and highlighting pathways enriched in either urban (n=105) or rural (n=6) children. The error bars indicate the standard error of the β-coefficients.

Supplementary Figure 7

a

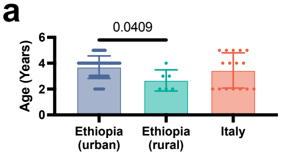

b

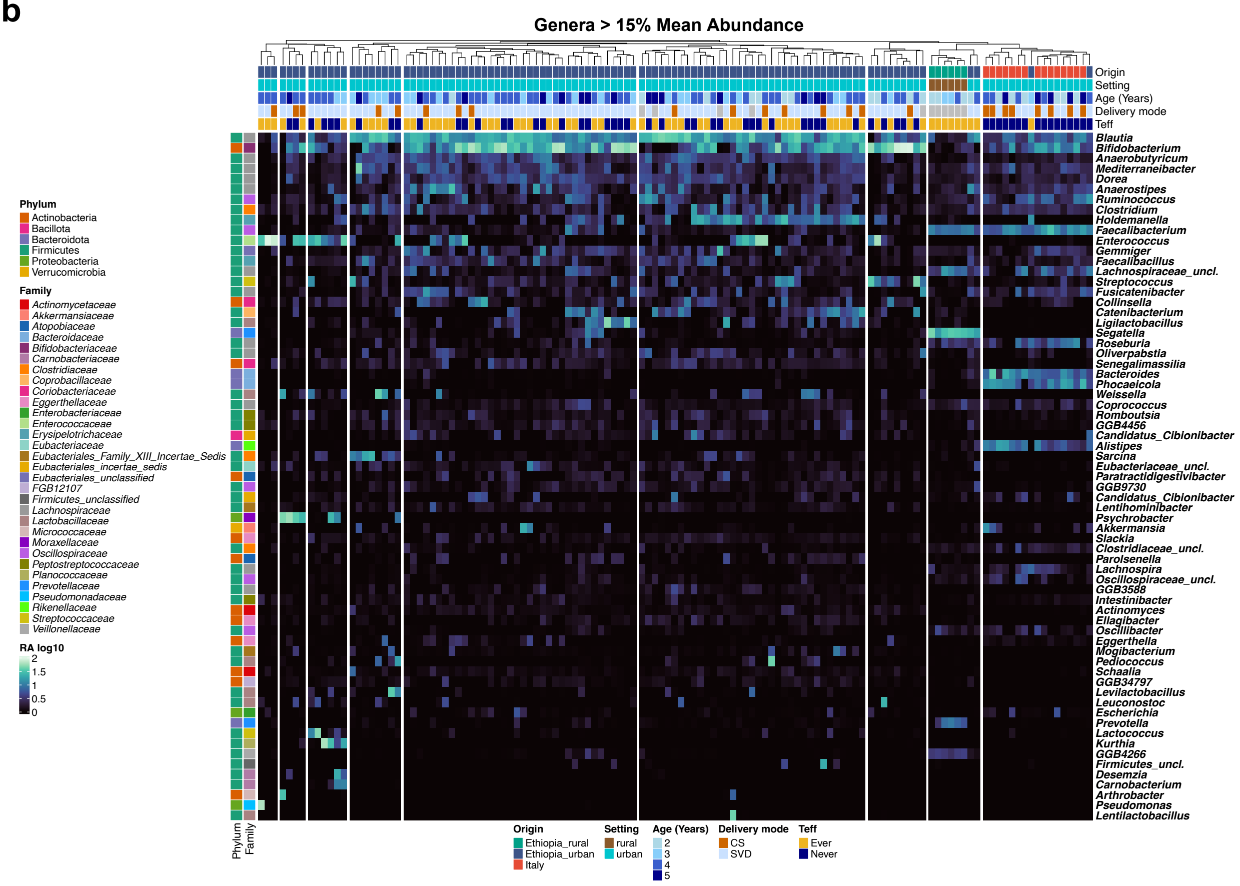

c

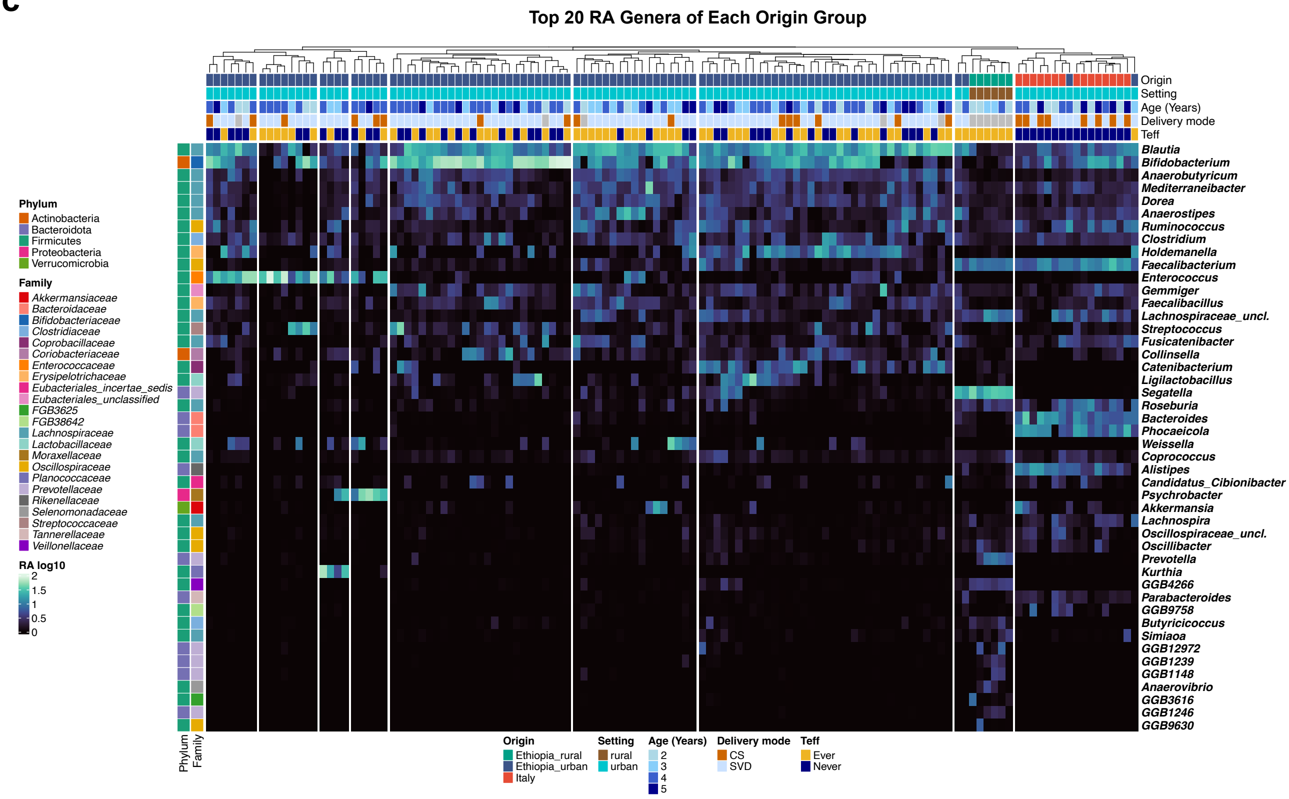

Supplementary Figure 7. Urbanization impacts gut microbial composition across cohorts. (a) Bar plot of age of children across the three cohorts (means +/- SD are shown). Differences between groups were assessed using Kruskal-Wallis tests with post-hoc pairwise comparisons. **(b)** Heatmap of genera relative abundances (log_{10}-transformed) across samples. Displayed are all genera with >15% mean relative abundance. Sample metadata, including origin, setting (urban/rural), age, delivery mode, and teff consumption, are annotated. Samples are clustered by similarity. **(c)** Heatmap of genera relative abundances (log_{10}-transformed) across samples. Displayed are the top 20 most abundant genera per cohort, sorted by overall mean abundance. Genera are annotated as row names, with phylum and family shown as left-side annotations. Sample metadata including origin, setting (urban/rural), age, delivery mode, and teff consumption are displayed. Samples are clustered by similarity. (n=105 urban Ethiopian, n=6 rural Ethiopian, n=16 Italian)

Supplementary Figure 8

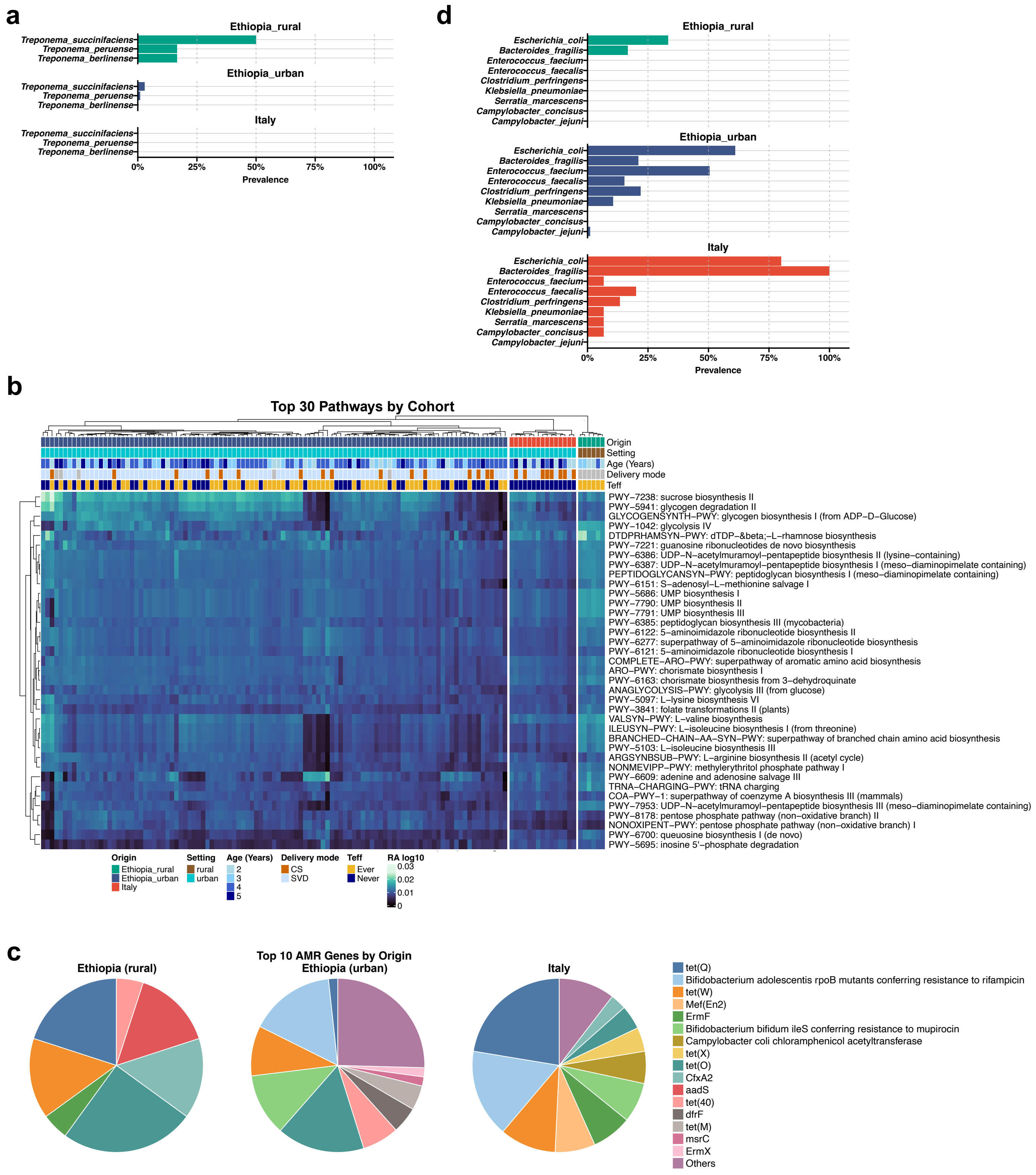

Supplementary Figure 8. Urbanization-associated differences in microbial pathways, antimicrobial resistance, and pathogen prevalence. (a) Prevalence of *Treponema berlinense*, *T. peruense* and *T. succinifaciens*, as detected by MetaPhlAn, stratified by cohort. Prevalence is the proportion of samples with non-zero relative abundance per species. **(b)** Heatmap of pathway relative abundances (log_{10}-transformed) across samples. Displayed are the top 30 most abundant pathways per cohort. Sample metadata, including origin, setting (urban/rural), age, delivery mode, and teff consumption are annotated. Samples and pathways are clustered by similarity. **(c)** Pie charts of the top 10 antimicrobial resistance (AMR) genes per cohort; genes with lower counts are grouped as "Others". **(d)** Prevalence of

selected potentially pathogenic species detected by MetaPhlAn, stratified by cohort. Prevalence was calculated as the proportion of samples in which each species had non-zero relative abundance. (n=105 urban Ethiopian, n=6 rural Ethiopian, n=16 Italian)
