# Supplementary material for: Gut microbial signatures expose the westernized lifestyle of urban Ethiopian children
Source: Commun Biol. 2026 Jan 31;9:346. doi: 10.1038/s42003-026-09639-2 (PMC12963580; doi:10.1038/s42003-026-09639-2)
Supplement: Supplementary file 3 — Description of Additional Supplementary Files (19.01.26) [file 42003_2026_9639_MOESM3_ESM.pdf]

File Name: Supplementary Data 1
Description: Overview of all cohorts and subsamples included in the study, including basic statistics (age distribution, delivery mode, breastfeeding status). The table also lists the two external cohorts obtained from published literature that were used for comparative analyses.

File Name: Supplementary Data 2
Description: Results of PERMANOVA and dispersion testing for categorical factors (Hp status, breastfeeding, delivery mode, age, stunting, family size, latrine in home, number of rooms in home, and Teff consumption) based on 16S rRNA gene sequencing data. Bray-Curtis dissimilarities were obtained from the qiime2 core-metrics pipeline. PERMANOVA was performed with adonis2 from the vegan R package (v2.6-10) using 999 permutations. The table reports R^2 values, ANOVA-based p-values, and permutation-based p-values for each factor. Group dispersion was tested using betadisper followed by permutest (999 permutations).

File Name: Supplementary Data 3
Description: Results of association tests between alpha-diversity metrics from 16S rRNA gene sequencing (imported from the QIIME 2 core-metrics output) and metadata variables. Two-level factors were tested with Wilcoxon rank-sum tests, while factors with more than two levels were analysed using Kruskal-Wallis tests followed by Dunn's post-hoc comparisons. For multi-level variables, post-hoc pairwise Wilcoxon tests were also applied where relevant. P-values within each factor were adjusted using the Benjamini-Hochberg procedure. Empty strings in metadata variables were treated as missing values and excluded per test. The table summarises test statistics and corrected p-values for all factors.

File Name: Supplementary Data 4
Description: Association of microbial alpha-diversity (Shannon index from 16S rRNA gene sequencing, QIIME 2 output) with delivery mode in children aged 2-5 years. Shannon indices were divided into six equal-count bins using the cut_number function in ggplot2 (R), and the proportion of C-section births was calculated within each bin and age group. Logistic regression models were fitted with delivery mode (C-section vs. vaginal delivery) as the outcome and age as a covariate. In the binned model, Shannon diversity was included as a categorical predictor (six bins, lowest bin as reference), with odds ratios, 95% confidence intervals, and a likelihood ratio χ^2 test. A second model treated Shannon diversity as a continuous predictor, yielding an age-adjusted odds ratio per unit increase and Wald test p-value. The table reports all model estimates and test results.

File Name: Supplementary Data 5
Description: Association of bacterial genus-level abundances with alpha-diversity and age based on 16S rRNA gene sequencing data. Amplicon sequence variant (ASV) tables and taxonomic assignments were imported from QIIME 2 artifacts, with raw counts transformed to counts per million (CPM) using edgeR (pseudocount of 1 added). CPM values were summed per genus to obtain sample-level abundances. Shannon diversity values were divided into six bins, and linear regression models were fitted for each genus with CPM as the outcome, Shannon diversity (categorical, 6 bins) and age (continuous) as predictors. Extracted outputs include the effect of age (estimate, standard error, p-value) and the overall effect of Shannon diversity (F-test across categories). Results from analogous models treating Shannon diversity as a continuous variable, as well as results from a Shannon-bin sensitivity analysis, are also reported.

File Name: Supplementary Data 6
Description: Summary statistics for shotgun metagenomic sequencing and processing. The table reports sequencing read counts per sample, percentages of host read removal, and contig assembly statistics.

File Name: Supplementary Data 7
Description: Results of PERMANOVA and dispersion testing for categorical factors based on shotgun metagenomic data. Bray-Curtis dissimilarities were derived from species-level MetaPhlAn4 profiles, calculated using the vegdist function from the vegan R package (v2.6-10). Analyses were performed with adonis2 (PERMANOVA) and betadisper/permutest (dispersion testing) with 999 permutations. The table reports R^2 values, ANOVA-based p-values, and permutation-based p-values for all tested factors.

File Name: Supplementary Data 8
Description: Results of association tests between alpha-diversity metrics derived from shotgun metagenomic species-level profiles and metadata variables. Species-level relative abundance profiles were obtained from MetaPhlAn outputs, retaining rows annotated at species level ("|s__") and excluding strain-level features ("|t__"). Alpha-diversity was quantified using Shannon and Simpson indices (diversity function, vegan R package) and observed richness was defined as the number of non-zero species per sample. Associations with metadata variables were tested using Wilcoxon rank-sum or Kruskal-Wallis tests depending on factor levels, with post-hoc comparisons and Benjamini-Hochberg correction applied as for the 16S rRNA gene sequencing data. The table reports test statistics and corrected p-values for all factors.

File Name: Supplementary Data 9
Description: Associations between presence or counts of species-level genome bins (SGBs) and Teff consumption. Binary indicators for each sample were generated for the presence (≥1) or absence (0) of unknown (uSGB) or known (kSGB) bins. Associations with Teff consumption (Ever vs. Never) were tested using generalised linear models (GLMs, stats R package), reporting odds ratios (ORs) with 95% confidence intervals and p-values. In addition, per-sample counts of distinct uSGBs and kSGBs were calculated and tested for association with Teff consumption using negative binomial regression models (glm.nb, MASS R package). Rate ratios (RRs) with 95% Wald confidence intervals and p-values are reported.

File Name: Supplementary Data 10
Description: Results of Fisher's exact tests assessing whether Teff consumption frequency differed by age group. Primary analyses compared age groups using Fisher's exact test. Because only one child in the 5-year-old group reported Teff consumption, a 2×2 Fisher's exact test was also performed contrasting 5-year-olds with all younger children (2-4 years). Effect sizes are reported as odds ratios with 95% confidence intervals.

File Name: Supplementary Data 11
Description: Results of differential abundance testing of COG categories between Teff consumers and non-consumers. Relative abundances were compared using Wilcoxon rank-sum tests. P-values were adjusted for multiple testing with the Benjamini-Hochberg FDR procedure, and categories with FDR < 0.05 were considered significant.

File Name: Supplementary Data 12
Description: Results of linear models testing whether Teff consumption influenced similarity to rural microbiomes. Bray-Curtis distances from each sample to the rural centroid (species-level relative abundances) were calculated. Models included Teff consumption (ever/never), age, and origin as predictors. A subgroup analysis restricted to urban children tested whether Teff consumption shifted them toward the rural centroid. The table reports model coefficients, confidence intervals, and p-values.

File Name: Supplementary Data 13
Description: Results of association analyses between AMR gene counts and metadata variables. RGI output tables were filtered to retain only high-confidence hits (≥100 supporting reads and ≥80% breadth of coverage). For each sample, the total number of AMR genes detected was calculated. Group comparisons across metadata categories (age, breastfeeding, delivery mode, Teff consumption, IgA-coating bins, household variables, and *H. pylori* status) were performed using Wilcoxon rank-sum tests for binary factors and Kruskal-Wallis tests for multi-level factors. P-values were adjusted using the Benjamini-Hochberg procedure.

File Name: Supplementary Data 14
Description: Source data for all figures
