# Supplementary material for: Gut microbial signatures expose the westernized lifestyle of urban Ethiopian children
Source: Commun Biol. 2026 Jan 31;9:346. doi: 10.1038/s42003-026-09639-2 (PMC12963580; doi:10.1038/s42003-026-09639-2)
Supplement: Supplementary file 4 — Reporting Summary [file 42003_2026_9639_MOESM4_ESM.pdf]

nature portfolio

Corresponding author(s): Anne Müller, Lydia Kirsche

Last updated by author(s): Jan 13, 2026

Reporting Summary

Nature Portfolio wishes to improve the reproducibility of the work that we publish. This form provides structure for consistency and transparency in reporting. For further information on Nature Portfolio policies, see our Editorial Policies and the Editorial Policy Checklist.

Statistics

For all statistical analyses, confirm that the following items are present in the figure legend, table legend, main text, or Methods section.

n/a	Confirmed	
☐	☒	The exact sample size (*n*) for each experimental group/condition, given as a discrete number and unit of measurement
☐	☒	A statement on whether measurements were taken from distinct samples or whether the same sample was measured repeatedly
☐	☒	The statistical test(s) used AND whether they are one- or two-sided<br>*Only common tests should be described solely by name; describe more complex techniques in the Methods section.*
☐	☒	A description of all covariates tested
☐	☒	A description of any assumptions or corrections, such as tests of normality and adjustment for multiple comparisons
☐	☒	A full description of the statistical parameters including central tendency (e.g. means) or other basic estimates (e.g. regression coefficient) AND variation (e.g. standard deviation) or associated estimates of uncertainty (e.g. confidence intervals)
☐	☒	For null hypothesis testing, the test statistic (e.g. *F*, *t*, *r*) with confidence intervals, effect sizes, degrees of freedom and *P* value noted<br>*Give P values as exact values whenever suitable.*
☒	☐	For Bayesian analysis, information on the choice of priors and Markov chain Monte Carlo settings
☒	☐	For hierarchical and complex designs, identification of the appropriate level for tests and full reporting of outcomes
☐	☒	Estimates of effect sizes (e.g. Cohen's *d*, Pearson's *r*), indicating how they were calculated

Our web collection on statistics for biologists contains articles on many of the points above.

Software and code

Policy information about availability of computer code

Data collection	Cytek SpectroFlo (v.3.3.0) was used to collect the flow cytometry data.
Data analysis	GraphPad Prism v10.5.0<br>Trimmomatic (V.0.39)<br>metaSPAdes (mode v3.15.5)<br>CheckM (v1.2.2)<br>Metabat2 (v 2:2.15)<br>MetaPhlAn4 (v 4.1.0)<br>HUMAnN3 (v 3.9)<br>QIIME2 (version 2025.7) with q2-sapienns (v2024.2.0)<br>vegan: Community Ecology Package (v 2.6-10)<br>PhyloPhlAn (v3.1.68)<br>Interactive Tree of Life (iTOL, v7.2)<br>prodigal (V2.6.3)<br>diamond (V2.0.11)<br>eggNOG mapper (emapper-2.1.12)<br>Resistance gene Identifier package (RGI, v6.0.0)

For manuscripts utilizing custom algorithms or software that are central to the research but not yet described in published literature, software must be made available to editors and reviewers. We strongly encourage code deposition in a community repository (e.g. GitHub). See the Nature Portfolio guidelines for submitting code & software for further information.

Data

Policy information about availability of data

All manuscripts must include a data availability statement. This statement should provide the following information, where applicable:

- Accession codes, unique identifiers, or web links for publicly available datasets
- A description of any restrictions on data availability
- For clinical datasets or third party data, please ensure that the statement adheres to our policy

Source data will be available upon publication in the National Center for Biotechnology Information Sequence Read Archive (SRA) under accession number: PRJNA1345963.

Research involving human participants, their data, or biological material

Policy information about studies with human participants or human data. See also policy information about sex, gender (identity/presentation), and sexual orientation and race, ethnicity and racism.

Reporting on sex and gender	A total of 207 participants were included in the study (105 female, 102 male). Sex and gender were reported by the participants' parents. We ensured a balanced distribution of sexes across the study population.
Reporting on race, ethnicity, or other socially relevant groupings	The children included in this study were primarily from the region around Adama, Ethiopia. Additional cultural or ethnic differences were not reported. Other socially relevant groupings were not assessed as part of the present study.
Population characteristics	The study population consisted of children aged 2 to 5 years, with a mean age of 3.7 years at the time of sampling. The average family size was 4.08 members (± 1.16 SD).
Recruitment	The birth cohort study from which the samples were derived was conducted in Adama City, Ethiopia, between April 2018 and December 2022. Recruitment took place at one public hospital and two primary health centres. Most participants were urban residents, with relatively better access to utilities and primarily engaged in small-scale trade.
Ethics oversight	The study was approved by the National Ethical Review Board of Ethiopia (Ref. No. 3.10/16/2018).

Note that full information on the approval of the study protocol must also be provided in the manuscript.

Field-specific reporting

Please select the one below that is the best fit for your research. If you are not sure, read the appropriate sections before making your selection.

☒ Life sciences ☐ Behavioural & social sciences ☐ Ecological, evolutionary & environmental sciences

For a reference copy of the document with all sections, see nature.com/documents/nr-reporting-summary-flat.pdf

Life sciences study design

All studies must disclose on these points even when the disclosure is negative.

Sample size	No sample size calculations were performed as part of the study. The number of individuals enrolled in the study was based on the availability.
Data exclusions	No data were excluded.
Replication	To ensure reproducibility in flow cytometry experiments, fluorescence minus one (FMO) and unstained controls were included. For sequencing, control samples were processed and sequenced in parallel to monitor data quality.
Randomization	Participants were not assigned to experimental groups; therefore, randomization was not applicable in this study.
Blinding	All analysis was performed blinded.

Reporting for specific materials, systems and methods

We require information from authors about some types of materials, experimental systems and methods used in many studies. Here, indicate whether each material, system or method listed is relevant to your study. If you are not sure if a list item applies to your research, read the appropriate section before selecting a response.

Materials & experimental systems

n/a	Involved in the study
☐	☒ Antibodies
☒	☐ Eukaryotic cell lines
☒	☐ Palaeontology and archaeology
☒	☐ Animals and other organisms
☒	☐ Clinical data
☒	☐ Dual use research of concern
☒	☐ Plants

Methods

n/a	Involved in the study
☒	☐ ChIP-seq
☐	☒ Flow cytometry
☒	☐ MRI-based neuroimaging

Antibodies

Antibodies used

anti-human IgA antibody (Alexa Fluor 647, Jackson ImmunoResearch, # 109-605-011, polyclonal)
SYTO BC (Invitrogen, #S34855, Nucleic Acid Stain)

Validation

Both antibodies used for flow cytometry were validated using fluorescence minus one (FMO) controls. Validation statements and specifications are available on the respective manufacturers' websites.

Plants

Seed stocks

Report on the source of all seed stocks or other plant material used. If applicable, state the seed stock centre and catalogue number. If plant specimens were collected from the field, describe the collection location, date and sampling procedures.

Novel plant genotypes

Describe the methods by which all novel plant genotypes were produced. This includes those generated by transgenic approaches, gene editing, chemical/radiation-based mutagenesis and hybridization. For transgenic lines, describe the transformation method, the number of independent lines analyzed and the generation upon which experiments were performed. For gene-edited lines, describe the editor used, the endogenous sequence targeted for editing, the targeting guide RNA sequence (if applicable) and how the editor was applied.

Authentication

Describe any authentication procedures for each seed stock used or novel genotype generated. Describe any experiments used to assess the effect of a mutation and, where applicable, how potential secondary effects (e.g. second site T-DNA insertions, mosiacism, off-target gene editing) were examined.

Flow Cytometry

Plots

Confirm that:

☐ The axis labels state the marker and fluorochrome used (e.g. CD4-FITC).

☐ The axis scales are clearly visible. Include numbers along axes only for bottom left plot of group (a 'group' is an analysis of identical markers).

☐ All plots are contour plots with outliers or pseudocolor plots.

☒ A numerical value for number of cells or percentage (with statistics) is provided.

Methodology

Sample preparation

Faecal samples were stored frozen until processing, which was performed as described in the Methods section of the manuscript.

Instrument

Flow cytometry data were acquired using the Cytek Aurora (5-laser) system.

Software

Flow cytometry data were acquired using SpectroFlo software (version 3.3.0) and analysed using FlowJo.

Cell population abundance

Samples were not sorted.

Gating strategy

Samples were first gated on the SytoBC^{+} population to distinguish bacteria from debris. Subsequently, events were gated on IgA^{+} to determine the fraction of IgA-coated bacteria.

☒ Tick this box to confirm that a figure exemplifying the gating strategy is provided in the Supplementary Information.
